# A Simple, Rapid and Mild One Pot Synthesis of Benzene Ring Acylated and Demethylated Analogues of Harmine under Solvent-free Conditions

**DOI:** 10.3390/molecules1301584

**Published:** 2008-08-06

**Authors:** Sabira Begum, Syed Nawazish Ali, Farhat, Syed Imran Hassan, Bina S. Siddiqui

**Affiliations:** H. E. J. Research Institute of Chemistry, International Center for Chemical and Biological Sciences, University of Karachi, Karachi-75270, Pakistan; E-mails: nawazishalii@yahoo.com (Syed Nawazish Ali); farhatzubair@yahoo.com (Farhat); imranhassan2@yahoo.com (Syed Imran Hassan); bina@comsats.net.pk (Bina S. Siddiqui)

**Keywords:** Solvent-free reactions, Friedel-Crafts acylation, *β*-carboline, harmine, acylharmine, demethylation

## Abstract

A simple, rapid, solvent-free, room temperature one pot synthesis of benzene ring acylated and demethylated analogues of harmine using acyl halides/acid anhydrides and AlCl_3_ has been developed. Eight different acyl halides/acid anhydrides were used in the synthesis. The resulting mixture of products was separated by column chromatography to afford 10- and 12-monoacyl analogues, along with 10,12-diacyl-11-hydroxy products. In five cases the corresponding 10-acyl-11-hydroxy analogues were also obtained. Yields from the eight syntheses (29 products in total) were in the 6-34% range and all compounds were fully characterized.

## Introduction

Solvent-free chemical syntheses have recently received much attention. These processes are not only environmentally benign, but also economically beneficial because toxic wastes can be minimized or eliminated, so the costs of waste treatment are also reduced. An additional attractive feature is their operational simplicity. Thus, there are now over 500 published papers describing solvent-free reactions giving quantitative yields on the gram and kilogram scale that support the effectiveness of the method [1,2,3,4].

Friedel-Crafts acylation is one of the most important protocols for the formation of C-C bonds between aromatic rings and aliphatic moieties. This method generally requires addition of a Lewis acid catalyst such as AlCl_3_ to a well-stirred mixture of an alkyl or acyl halide and an aromatic compound under inert conditions. Usually, these reactions require high temperatures, long reaction times and tedious workups [5,6]. Recently, Friedel-Crafts acylation of alkoxybenzenes was achieved efficiently by a reaction with aliphatic acid anhydrides in the presence of catalytic amounts of aluminum hydrogensulfate, Al(HSO_4_)_3_, both in nitromethane and under solvent-free conditions, but these reactions required high temperaures (70 ^o^C) for completion [7]. Although the acetylation of aryl ethers using acetic anhydride in the presence of zeolites under mild conditions in a solvent-free system gave the corresponding *para*-acetylated products in high yields, these reactions also required high temperatures (120 ^o^C) and occurred only in activated aromatic compounds [8]. The use of metal triflates in ionic liquids for Friedel-Crafts acylation have also been reported, but it is again limited to highly activated substrates and also requires high temperatures [9].

The *β*-carboline alkaloids, one of which is harmine, are very important natural products due to their interesting chemistry, pharmacological importance and therapeutic potential. They posses anti-tumor, anti-HIV and other important biological activities [10,11,12]. Recently, we have reported the Friedel-Crafts acylation of *N*-acetyltetrahydroharmine under solvent-free conditions, which resulted in the synthesis of a series of its 10-acyl and 12-acyl analogues in high overall yields [13]. Here we would like to present a study of the reaction of the Friedel-Crafts reagents (acyl halides/acid anhydrides and AlCl_3_) with harmine (7-MeO-1-Me-9H-pyrido[3,4-b]-indole, **1**), another *β*-carboline alkaloid, at room temperature and under solvent-free conditions. The harmine used in these investigations was isolated from *Peganum harmala* following a method reported earlier [14]. Eight different syntheses were carried out, which yielded 29 different products. None of these products had previously been reported. After separation by column chromatography the reaction mixtures gave 10-acyl (**2-9**), 12-acyl (**10-17**), 10-acyl-11-hydroxy (**18-22**) and 10,12-diacyl-11-hydroxy (**23-30**) derivatives of harmine (**1**). All compounds were fully characterized. 

## Results and Discussion

The solid phase reaction of harmine (**1**) with different acylating agents (acetic anhydride, propionyl chloride, butyric anhydride, *iso*-butyric anhydride, valeryl chloride, hexanoyl chloride, heptanoyl chloride and capryloyl chloride) in the presence of AlCl_3_ yielded mono acyl derivatives without demethylation as well as mono and diacyl derivatives with demethylation. The acylation took place at the carbons *ortho* to the methoxy group (C-10 acylated products **2-9**) and (C-12 acylated products **10-17**). Acylation with demethylation of the C-11 OCH_3_ group yielded both monoacyl (C-10-acyl,11-hydroxy **18-22**) and diacyl (C-10,12-diacyl,11-hydroxy **23-30**) products.


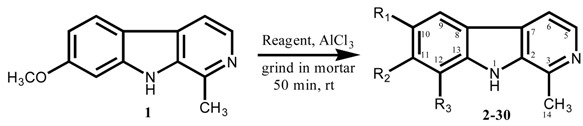


**Table 1:** Friedel-Crafts acylation of **1** under solvent-free conditions.

Entry	Reagents	Products	R_1_	R_2_	R_3_	Yield^a^ (%)
**A**	(CH_3_CO)_2_O	**2**	^1'^CO-^2'^CH_3_	OCH_3_	H	14.4
		**10**	H	OCH_3_	^1'^CO-^2'^CH_3_	12.5
		**23**	^1'^CO-^2'^CH_3_	OH	^1''^CO-^2''^CH_3_	12.7
**B**	CH_3_CH_2_COCl	**3**	^1'^CO-^2'^CH_2_-^3'^CH_3_	OCH_3_	H	33.6
		**11**	H	OCH_3_	^1'^CO-^2'^CH_2_-^3'^CH_3_	12.7
		**18**	^1'^CO-^2'^CH_2_-^3'^CH_3_	OH	H	12.8
		**24**	^1'^CO-^2'^CH_2_-^3'^CH_3_	OH	^1''^CO-^2''^CH_2_-^3''^CH_3_	14.6
**C**	(CH_3_-CH_2_-CH_2_-CO)_2_O	**4**	^1'^CO-(CH_2_)_2_-^4'^CH_3_	OCH_3_	H	32.1
		**12**	H	OCH_3_	^1'^CO-(CH_2_)_2_-^4'^CH_3_	12.5
		**19**	^1'^CO-(CH_2_)_2_-^4'^CH_3_	OH	H	7.0
		**25**	^1'^CO-(CH_2_)_2_-^4'^CH_3_	OH	^1''^CO-(CH_2_)_2_-^4''^CH_3_	9.7
**D**	((CH_3_)_2_CH-CO)_2_O	**5**	^1'^CO-^2'^CH(CH_3_)_2_	OCH_3_	H	20.7
		**13**	H	OCH_3_	^1'^CO-^2'^CH(CH_3_)_2_	7.9
		**20**	^1'^CO-^2'^CH(CH_3_)_2_	OH	H	20.2
		**26**	^1'^CO-^2'^CH(CH_3_)_2_	OH	^1''^CO-^2''^CH(CH_3_)_2_	7.9
**E**	CH_3_-(CH_2_)_3_-CO-Cl	**6**	^1'^CO-(CH_2_)_3_-^5'^CH_3_	OCH_3_	H	18.8
		**14**	H	OCH_3_	^1'^CO-(CH_2_)_3_-^5'^CH_3_	10.1
		**27**	^1'^CO-(CH_2_)_3_-^5'^CH_3_	OH	^1''^CO-(CH_2_)_3_-^5''^CH_3_	14.8
**F**	CH_3_-(CH_2_)_4_-CO-Cl	**7**	^1'^CO-(CH_2_)_4_-^6'^CH_3_	OCH_3_	H	20.0
		**15**	H	OCH_3_	^1'^CO-(CH_2_)_4_-^6'^CH_3_	12.6
		**21**	^1'^CO-(CH_2_)_4_-^6'^CH_3_	OH	H	10.2
		**28**	^1'^CO-(CH_2_)_4_-^6'^CH_3_	OH	^1''^CO-(CH_2_)_4_-^6''^CH_3_	13.5
**G**	CH_3_-(CH_2_)_5_-CO-Cl	**8**	^1'^CO-(CH_2_)_5_-^7'^CH_3_	OCH_3_	H	26.8
		**16**	H	OCH_3_	^1'^CO-(CH_2_)_5_-^7'^CH_3_	11.8
		**22**	^1'^CO-(CH_2_)_5_-^7'^CH_3_	OH	H	5.9
		**29**	^1'^CO-(CH_2_)_5_-^7'^CH_3_	OH	^1''^CO-(CH_2_)_5_-^7''^CH_3_	14.1
**H**	CH_3_-(CH_2_)_6_-CO-Cl	**9**	^1'^CO-(CH_2_)_6_-^8'^CH_3_	OCH_3_	H	31.1
		**17**	H	OCH_3_	^1'^CO-(CH_2_)_6_-^8'^CH_3_	11.5
		**30**	^1'^CO-(CH_2_)_6_-^8'^CH_3_	OH	^1''^CO-(CH_2_)_6_-^8''^CH_3_	12.7

*^a^* Isolated yields of the products.

These reaction products were separated by silica gel column chromatography. It was noted that 10-acyl derivatives were obtained as the major products. The remaining derivatives were also obtained in acceptable yields (Table 1). No 10-acyl-11-hydroxy derivatives could be isolated in the present work from the products of the reaction with acetic anhydride (entry A), valeryl chloride (entry E) and capryloyl chloride (entry H). All these harmine derivatives are reported for the first time. The 10,12-diacyl-11-hydroxy products may be formed from 10-acyl-11-hydroxy compounds through direct Friedel-Crafts acylation at C-12, or may be obtained after esterification of phenolic OH, through Fries rearrangement in the presence of AlCl_3_ [15]. The demethylation of 11-OCH_3_ in the presence of Lewis acid (AlCl_3_) may be due to the electron withdrawing effect of the 10-acyl group and the pyridine ring of harmine. The resulting phenolic OH facilitates the introduction of second acyl group in the benzene ring and considerable quantities of 10,12-diacyl-11-hydroxy derivatives of harmine were obtained under these solvent-free conditions. Earlier it has been reported that phenols do not react satisfactorily with Friedel-Crafts reagent in solvents because of their reaction with Lewis acids (Ar-OH + AlCl_3_ ➔ Ar-O-AlCl_2_ + HCl) resulting in compounds which are usually only sparingly soluble in the reaction medium and hence slow down the reactions [16]. It is important to note that under the same reaction conditions Ghiaci and Asghari [17] reported *O*-acylated phenols but in the present studies no *O*-acylated product was obtained. Further, under these reaction conditions no acylation of the pyridine ring of **1** occurred.

**Figure 1:**
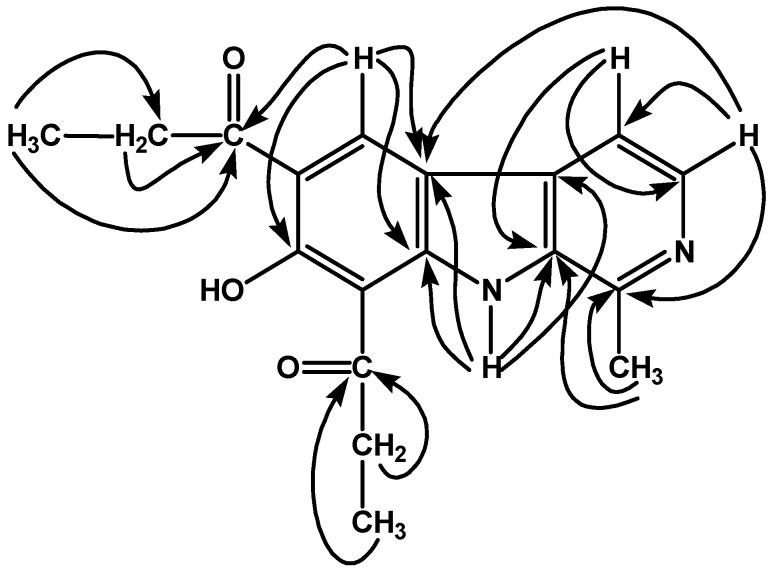
Significant HMBC (^1^H ➔ ^13^C) interactions of 10,12-dipropionyl-11-hydroxy (**24**) analogue of **1**.

The derivatives have been characterized by spectral studies, including IR, UV, EIMS, HREIMS, 1D (^1^H-NMR and ^13^C-NMR; Broad Band decoupled, DEPT), and 2D-NMR (COSY-45, TOCSY, HMQC and HMBC) (See Experimental, Table 2 and Table 3) and comparison of the spectral data with reported values of similar compounds [13,18,19]. For the 10-acyl analogues (see compound **3** in Table 2), the characteristic H-9 and H-12 peaks appeared in the ^1^H-NMR spectra as one-proton singlets at ~ δ 8.5 and 7.1, respectively, confirming the substitution at C-10. The ^1^H-NMR spectra of 12-acyl analogues (compound **11**) characteristically showed two sets of one-proton doublets at ~ δ 8.2 and 6.9, assigned to H-9 and H-10 respectively, showing the substitution on C-12. For 10-acyl demethylated analogues (compound **18**), the characteristic peaks of H-9 and H-12 appeared as one-proton singlets at ~ δ 8.8 and 6.9, respectively, whereas for 10,12-diacyl demethylated analogues (compound **24**), the characteristic H-9 peak appeared as a one-proton singlet at ~ δ 8.7 and a peak for chelated phenolic OH appeared at ~ δ 14 in CDCl_3_ in the ^1^H-NMR spectra. The demethylation was also confirmed from their high resolution mass spectral data (See Experimental). The ^13^C-NMR signals of quaternary carbons were particularly assigned on the basis of HMBC connectivities observed for these carbons with various protons. The typical HMBC connectivities of various protons with different carbons are also shown in Figure 1 for compound **24**.

## Conclusions

In this study new benzene ring substituted and demethylated derivatives of harmine were obtained for the first time in one-pot using acyl halides/acid anhydride and AlCl_3_ under solvent-free conditions at room temperature after short reaction times. The derivatives obtained in these studies are being screened for potential biological activities such as anti-cancer, anti-bacterial, anti-fungal, CNS and anti-hypertensive properties. The results will be reported in due course. Further the *β*-carboline alkaloids possessing acyl substituents on C-10 and C-12 are rare in nature, hence the spectral data presented in this communication should be a useful contribution to the structural elucidation of related natural products.

## Experimental

### General

Melting points were determined using a Buchi-535 melting point apparatus and are uncorrected. Ultraviolet spectra were measured on a Hitachi-3200 spectrophotometer. Infrared spectra were recorded on a Bruker VECTOR 22 spectrophotometer. The ^1^H- and ^13^C-NMR spectra were recorded at 400 and 100 MHz, respectively, on a Bruker Avance 400 spectrometer. Mass spectra were run on a Jeol JMS-HX110 (high resolution, E.I. probe, 70 eV) and a Varian MAT-312 (low resolution, E.I. probe, 70 eV) instrument.

### Solvent-Free Friedel-Crafts Acylation: General Procedure

A mixture of harmine (**1**, 100 mg), acylating agents (3.5 mL) and anhydrous AlCl_3_ (400 mg) was thoroughly ground in an agate mortar with a pestle for 50 min in a fume cupboard. The reaction mixture was then kept at room temperature for 1 hour and poured into crushed ice, made basic with 30% aqueous NH_4_OH and extracted with ethyl acetate. The ethyl acetate layer gave a solid mass after washing, drying (Na_2_SO_4_) and removal of solvents under reduced pressure. Further workup using column chromatography (silica gel; Merck 9385; CHCl_3_-MeOH, in increasing order of polarity from 9.95:0.05 to 9.5:0.5) afforded 10,12-diacyl,11-hydroxy (**23-30**; TLC data: R_f_ ~ 0.90, 9.5:0.5 CHCl_3_-MeOH), 12-acyl (**10-17**; TLC data: R_f_ ~ 0.86, 9.5:0.5 CHCl_3_-MeOH), 10-acyl,11-hydroxy (**18-22**; TLC data: R_f_ ~ 0.70, 9.5:0.5 CHCl_3_-MeOH ) and 10-acyl (**2-9**; TLC data: R_f_ ~ 0.61, 9.5:0.5 CHCl_3_-MeOH) derivatives, respectively, as colorless crystalline solids, in moderate yields (Table 1).

*10-Acetyl-11-methoxy-3-methyl-β-carboline* (**2**). 17.3 mg; mp: 265-266 ^o^C; IR (CHCl_3_) ν_max_ cm^-1^: 3263.2 (indole N-H) 2962.6, 2847.8 (C-H), 1656.4 (ketone C=O), 1606.1, 1524.2, 1457.0 (aromatic C=C), 1162.3 (C-O); UV (MeOH) λ_max_ nm: 330.1, 270.1, 229.7; ^1^H-NMR (CD_3_OD): δ 8.18 (1H, d, *J =* 5.7 Hz, H-5), 8.05 (1H, d, *J =* 5.7 Hz, H-6), 8.46 (1H, s, H-9), 7.14 (1H, s, H-12), 2.86 (3H, s, H-14), 2.98 (3H, s, H-2′), 4.01 (3H, s, OCH_3_); ^13^C-NMR (CD_3_OD): δ 136.5 (C-2), 142.1 (C-3), 139.3 (C-5), 113.2 (C-6), 116.0 (C-7), 130.8 (C-8), 125.9 (C-9), 123.9 (C-10), 161.6 (C-11), 94.5 (C-12), 146.6 (C-13), 19.6 (C-14), 204.6 (C-1′), 32.4 (C-2′), 56.8 (OCH_3_); HREIMS (*m/z*): 254.1048 [C_15_H_14_N_2_O_2_ calcd. 254.1055]; EIMS *m/z* (%): 254 [M^+^] (100), 239 (94), 224 (36), 211 (4), 196 (28).

*10-Propionyl-11-methoxy-3-methyl-β-carboline* (**3**). 39.2 mg; mp: 268-269 ^o^C; IR (CHCl_3_) ν_max_ cm^-1^: 3264.2 (indole N-H), 2963.2, 2848.6 (C-H), 1656.1 (ketone C=O), 1604.1, 1525.1, 1453.2 (aromatic C=C), 1166.3 (C-O); UV (MeOH) λ_max_ nm: 329.6, 269.4, 230.2; ^1^H-NMR (CD_3_OD): Table 2; ^13^C-NMR (CD_3_OD): Table 3; HREIMS (*m/z*): 268.1202 [C_16_H_16_N_2_O_2_ calcd. 268.1212]; EIMS *m/z* (%): 268 [M^+^] (60), 253 (10), 239 (100), 224 (40), 211 (3), 196 (32).

*10-Butyryl-11-methoxy-3-methyl-β-carboline* (**4**). 42.7 mg; mp: 269-270 ^o^C; IR (CHCl_3_) ν_max_ cm^-1^: 3268.2 (indole N-H), 2927.1, 2854.2 (C-H), 1657.1 (ketone C=O), 1608.3, 1523.9, 1412.5 (aromatic C=C), 1152.1 (C-O); UV (MeOH) λ_max_ nm: 329.8, 269.8, 229.5; ^1^H-NMR (CD_3_OD): δ 8.18 (1H, d, *J =* 5.4 Hz, H-5), 8.04 (1H, d, *J =* 5.4 Hz, H-6), 8.48 (1H, s, H-9), 7.15 (1H, s, H-12), 2.88 (3H, s, H-14), 3.03 (2H, t, *J =* 7.3 Hz, H-2′), 1.72 (2H, sextet, *J =* 7.3 Hz, H-3′), 0.99 (3H, t, *J =* 7.3 Hz, H-4′), 4.03 (3H, s, OCH_3_); ^13^C-NMR (CD_3_OD): δ 136.3 (C-2), 142.5 (C-3), 139.7 (C-5), 113.6 (C-6), 116.1 (C-7), 130.9 (C-8), 125.7 (C-9), 123.8 (C-10), 161.5 (C-11), 94.6 (C-12), 146.4 (C-13), 19.0 (C-14), 204.3 (C-1′), 46.0 (C-2′), 18.6 (C-3′), 14.2 (C-4′), 56.8 (OCH_3_); HREIMS (*m/z*): 282.1360 [C_17_H_18_N_2_O_2_ calcd. 282.1368]; EIMS *m/z* (%): 282 [M^+^] (30), 267 (6), 239 (100), 224 (10), 211 (2), 196 (28). 

*10-Isobutyryl-11-methoxy-3-methyl-β-carboline* (**5**). 27.5 mg; mp: 273-274 ^o^C; IR (CHCl_3_) ν_max_ cm^-1^: 3252.2 (indole N-H), 2947.0, 2853.1 (C-H), 1656.2 (ketone C=O), 1607.5, 1524.7, 1456.5 (aromatic C=C), 1152.2 (C-O); UV (MeOH) λ_max_ nm: 330.0, 269.7, 229.5; ^1^H-NMR (CD_3_OD): δ 8.20 (1H, d, *J =* 5.5 Hz, H-5), 8.04 (1H, d, *J =* 5.5 Hz, H-6), 8.47 (1H, s, H-9), 7.14 (1H, s, H-12), 2.86 (3H, s, H-14), 3.60 (1H, septet, *J =* 6.8 Hz, H-2′), 1.13 (6H, d, *J =* 6.8 Hz, H-3′ and H-4′), 4.02 (3H, s, OCH_3_); ^13^C-NMR (CD_3_OD): δ 136.4 (C-2), 142.2 (C-3), 139.2 (C-5), 113.5 (C-6), 116.3 (C-7), 130.5 (C-8), 125.8 (C-9), 124.1 (C-10), 161.4 (C-11), 94.5 (C-12), 146.4 (C-13), 19.2 (C-14), 208.4 (C-1′), 40.1 (C-2′), 18.8 (C-3′ and C-4′), 56.5 (OCH_3_); HREIMS (*m/z*): 282.1357 [C_17_H_18_N_2_O_2_ calcd. 282.1368]; EIMS *m/z* (%): 282 [M^+^] (92), 239 (100), 224 (6), 211 (8), 196 (21). 

*10-Valeryl-11-hydroxy-3-methyl-β-carboline* (**6**). 26.3 mg; mp: 272-273 ^o^C; IR (CHCl_3_) ν_max_ cm^-1^: 3252.6 (indole N-H), 2928.0, 2860.2 (C-H), 1655.8 (ketone C=O), 1601.6, 1525.4, 1423.1 (aromatic C=C), 1152.8 (C-O); UV (MeOH) λ_max_ nm: 330.2, 269.8, 229.6; ^1^H-NMR (CD_3_OD): δ 8.18 (1H, d, *J =* 5.8 Hz, H-5), 8.08 (1H, d, *J =* 5.8 Hz, H-6), 8.45 (1H, s, H-9), 7.12 (1H, s, H-12), 2.87 (3H, s, H-14), 3.02 (2H, t, *J =* 7.3 Hz, H-2′), 1.66 (2H, quintet, *J =* 7.3 Hz, H-3′), 1.40 (2H, sextet, *J =* 7.3 Hz, H-4′), 0.94 (3H, t, *J =* 7.3 Hz, H-5′), 4.03 (3H, s, OCH_3_); ^13^C-NMR (CD_3_OD): δ 136.5 (C-2), 142.6 (C-3), 139.0 (C-5), 113.8 (C-6), 116.0 (C-7), 130.7 (C-8), 126.5 (C-9), 124.2 (C-10), 161.5 (C-11), 94.7 (C-12), 146.7 (C-13), 19.4 (C-14), 204.5 (C-1′), 44.2 (C-2′), 28.1 (C-3′), 23.5 (C-4′), 14.3 (C-5′), 56.6 (OCH_3_); HREIMS (*m/z*): 296.1512 [C_18_H_20_N_2_O_2_ calcd. 296.1525]; EIMS *m/z* (%): 296 [M^+^] (12), 267 (2), 254 (14), 239 (100), 224 (8), 211 (3), 196 (7).

*10-Hexanoyl-11-methoxy-3-methyl-β-carboline* (**7**). 29.3 mg; mp: 274-275 ^o^C; IR (CHCl_3_) ν_max_ cm^-1^: 3251.8 (indole N-H), 2925.8, 2856.4 (C-H), 1655.2 (ketone C=O), 1602.3, 1523.3, 1458.1 (aromatic C=C), 1168.1 (C-O); UV (MeOH) λ_max_ nm: 330.1, 269.8, 231.4; ^1^H-NMR (CD_3_OD): δ 8.19 (1H, d, *J =* 5.4 Hz, H-5), 8.03 (1H, d, *J =* 5.4 Hz, H-6), 8.45 (1H, s, H-9), 7.12 (1H, s, H-12), 2.85 (3H, s, H-14), 3.02 (2H, t, *J =* 7.4 Hz, H-2′), 1.68 (2H, quintet, *J =* 7.4 Hz, H-3′), 1.36 (4H, m, H-4′ and H-5′), 0.91 (3H, t, *J =* 7.4 Hz, H-6′), 4.01 (3H, s, OCH_3_); ^13^C-NMR (CD_3_OD): δ 137.1 (C-2), 142.7 (C-3), 139.1 (C-5), 113.7 (C-6), 116.2 (C-7), 130.6 (C-8), 125.8 (C-9), 124.0 (C-10), 161.6 (C-11), 94.5 (C-12), 146.5 (C-13), 19.6 (C-14), 204.8 (C-1′), 44.5 (C-2′), 25.7 (C-3′), 32.8 (C-4′), 23.6 (C-5′), 14.3 (C-6′), 56.3 (OCH_3_); HREIMS (*m/z*): 310.1670 [C_19_H_22_N_2_O_2_ calcd. 310.1681]; EIMS *m/z* (%) : 310 [M^+^] (8), 267 (4), 254 (18), 239 (100), 224 (8), 211 (2), 196 (7). 

*10-Heptanoyl-11-methoxy-3-methyl-β-carboline* (**8**). 40.9 mg; mp: 277-278 ^o^C; IR (CHCl_3_) ν_max_ cm^-1^: 3255.6 (indole N-H), 2923.5, 2858.1 (C-H), 1656.8 (ketone C=O), 1608.2, 1526.1, 1453.1 (aromatic C=C), 1150.2 (C-O); UV (MeOH) λ_max_ nm: 330.2, 269.3, 231.8; ^1^H-NMR (CD_3_OD): δ 8.21 (1H, d, *J =* 5.4 Hz, H-5), 8.05 (1H, d, *J =* 5.4 Hz, H-6), 8.47 (1H, s, H-9), 7.15 (1H, s, H-12), 2.87 (3H, s, H-14), 2.99 (2H, t, *J =* 7.2 Hz, H-2′), 1.66 (2H, m, H-3′), 1.32 (6H, m, H-4′, H-5′ and H-6′), 0.90 (3H, t, *J =* 7.2 Hz, H-7′), 3.99 (3H, s, OCH_3_); ^13^C-NMR (CD_3_OD): δ 136.9 (C-2), 142.5 (C-3), 139.3 (C-5), 113.5 (C-6), 116.3 (C-7), 130.6 (C-8), 126.0 (C-9), 124.3 (C-10), 162.0 (C-11), 94.7 (C-12), 146.7 (C-13), 19.7 (C-14), 204.1 (C-1′), 44.6 (C-2′), 24.2 (C-3′), 30.3 (C-4′), 32.2 (C-5′), 23.2 (C-6′), 14.6 (C-7′), 56.6 (OCH_3_); HREIMS (*m/z*): 324.1851 [C_20_H_24_N_2_O_2_ calcd. 324.1838]; EIMS *m/z* (%): 324 [M^+^] (14), 267 (5), 254 (22) 239 (100), 224 (8), 211 (3), 196 (9). 

*10-Capryloyl-11-methoxy-3-methyl-β-carboline* (**9**). 49.6 mg; mp: 280-281 ^o^C; IR (CHCl_3_) ν_max_ cm^-1^: 3250.2 (indole N-H), 2925.8, 2856.4 (C-H), 1657.1 (ketone C=O), 1607.8, 1521.8, 1454.8 (aromatic C=C), 1152.4 (C-O); UV (MeOH) λ_max_ nm: 330.8, 270.1, 232.2; ^1^H-NMR (CD_3_OD): δ 8.22 (1H, d, *J =* 5.6 Hz, H-5), 8.06 (1H, d, *J =* 5.6 Hz, H-6), 8.46 (1H, s, H-9), 7.13 (1H, s, H-12), 2.89 (3H, s, H-14), 3.04 (2H, t, *J =* 7.3 Hz, H-2′), 1.69 (2H, quintet, *J =* 7.3 Hz, H-3′), 1.35 (2H, quintet, *J =* 7.3 Hz, H-4′), 1.33 (6H, m, H-5′, H-6′ and H-7′), 0.88 (3H, t, *J =* 7.3 Hz, H-8′), 4.05 (3H, s, OCH_3_); ^13^C-NMR (CD_3_OD): δ 136.1 (C-2), 142.6 (C-3), 139.2 (C-5), 113.8 (C-6), 116.1 (C-7), 130.5 (C-8), 126.1 (C-9), 124.2 (C-10), 161.4 (C-11), 94.8 (C-12), 146.6 (C-13), 19.5 (C-14), 204.5 (C-1′), 43.9 (C-2′), 25.1 (C-3′)*, 29.8 (C-4′)**, 29.6 (C-5′)**, 31.9 (C-6′), 25.4 (C-7′)*, 14.5 (C-8′), 56.7 (OCH_3_) (*,** values may be interchanged); HREIMS (*m/z*): 338.1985 [C_21_H_26_N_2_O_2_ calcd. 338.1994]; EIMS *m/z* (%): 338 [M^+^] (6), 267 (5), 254 (21), 239 (100), 224 (10), 211 (8), 196 (6). 

*12-Acetyl-11-methoxy-3-methyl-β-carboline* (**10**). 15.0 mg; mp: 283-284 ^o^C; IR (CHCl_3_) ν_max_ cm^-1^: 3388.7 (indole N-H), 2922, 2848.7 (C-H), 1648.2 (ketone C=O), 1608.3, 1545.7, 1427.2 (aromatic C=C), 1151.6 (C-O); UV (MeOH) λ_max_ nm: 361.8, 321.3, 269.7, 232.5; ^1^H-NMR (CDCl_3_): δ 8.35 (1H, d, *J =* 5.3 Hz, H-5), 7.83 (1H, d, *J =* 5.3 Hz, H-6), 8.23 (1H, d, *J =* 8.6 Hz, H-9), 6.99 (1H, d, *J =* 8.6 Hz, H-10), 2.95 (3H, s, H-14), 2.78 (3H, s, H-2′), 4.09 (3H, s, OCH_3_), 11.17 (1H, br.s, indole N-H); ^13^C-NMR (CDCl_3_): δ 134.2 (C-2), 137.9 (C-3), 131.2 (C-5), 113.9 (C-6), 115.9 (C-7), 131.8 (C-8), 129.4 (C-9), 107.7 (C-10), 163.8 (C-11), 109.7 (C-12), 145.0 (C-13), 16.2 (C-14), 203.2 (C-1′), 33.6 (C-2′), 56.6 (OCH_3_); HREIMS (*m/z*): 254.1042 [C_15_H_14_N_2_O_2_ calcd. 254.1055]; EIMS *m/z* (%): 254 [M^+^] (96), 239 (100), 224 (31), 211 (3), 196 (33).

*12-Propionyl-11-methoxy-3-methyl-β-carboline* (**11**). 15.7 mg; mp: 284-285 ^o^C; IR (CHCl_3_) ν_max_ cm^-1^: 3388.2 (indole N-H), 2934.1, 2841.2 (C-H), 1648.7 (ketone C=O), 1607.2, 1545.7, 1472.1 (aromatic C=C), 1152.4 (C-O); UV (MeOH) λ_max_ nm: 361.4, 321.6, 269.4, 232.5; ^1^H-NMR (CD_3_OD): Table 2; ^13^C-NMR (CD_3_OD): Table 3; HREIMS (*m/z*): 268.1223 [C_16_H_16_N_2_O_2_ calcd. 268.1212]; EIMS *m/z* (%): 268 [M^+^] (46,), 253 (4), 239 (100), 224 (31), 211 (4), 196 (29). 

*12-Butyryl-11-methoxy-3-methyl-β-carboline* (**12**). 10.5 mg; mp: 287-288 ^o^C; IR (CHCl_3_) ν_max_ cm^-1^: 3390.1 (indole N-H) 2956.7, 2871.8 (C-H), 1647.9 (ketone C=O), 1610.3, 1544.5, 1434.9 (aromatic C=C), 1152.4 (C-O); UV (MeOH) λ_max_ nm: 361.2, 321.4, 269.8, 232.4 nm; ^1^H-NMR (CDCl_3_): δ 8.33 (1H, d, *J =* 5.4 Hz, H-5), 7.81 (1H, d, *J =* 5.4 Hz, H-6), 8.19 (1H, d, *J =* 8.7 Hz, H-9), 6.95 (1H, d, *J =* 8.7 Hz, H-10), 2.98 (3H, s, H-14), 3.14 (2H, t, *J =* 7.3 Hz, H-2′), 1.79 (2H, sextet, *J =* 7.3 Hz, H-3′), 1.03 (3H, t, *J =* 7.3 Hz, H-4′), 4.06 (3H, s, OCH_3_), 11.16 (1H, br.s, indole N-H); ^13^C-NMR (CDCl_3_): δ 134.4 (C-2), 138.1 (C-3), 131.0 (C-5), 113.9 (C-6), 116.0 (C-7), 131.9 (C-8), 129.3 (C-9), 107.5 (C-10), 163.6 (C-11), 109.8 (C-12), 145.0 (C-13), 16.4 (C-14), 203.3 (C-1′), 45.3 (C-2′), 18.0 (C-3′), 14.2 (C-4′), 56.9 (OCH_3_); HREIMS (*m/z*): 282.1377 [C_17_H_18_N_2_O_2_ calcd. 282.1368]; EIMS *m/z* (%): 282 [M^+^] (32), 267 (8), 239 (100), 224 (17), 211 (3), 196 (28). 

*12-Isobutyryl-11-methoxy-3-methyl-β-carboline* (**13**). 16.6 mg; mp: 290-291 ^o^C; IR (CHCl_3_) ν_max_ cm^-1^: 3384.1 (indole N-H), 2927.2, 2857.2 (C-H), 1648.9 (ketone C=O), 1614.2, 1544.7, 1460.6 (aromatic C=C), 1151.3 (C-O); UV (MeOH) λ_max_ nm: 361.3, 321.4, 269.4, 232.2; ^1^H-NMR (CDCl_3_): δ 8.34 (1H, d, *J =* 5.5 Hz, H-5), 7.80 (1H, d, *J =* 5.5 Hz, H-6), 8.21 (1H, d, *J =* 8.7 Hz, H-9), 6.98 (1H, d, *J =* 8.7 Hz, H-10), 2.96 (3H, s, H-14), 3.88 (1H, septet, *J =* 6.7 Hz, H-2′), 1.23 (6H, d, *J =* 6.7 Hz, H-3′ and H-4′), 4.08 (3H, s, OCH_3_), 11.21 (1H, br.s, indole N-H); ^13^C-NMR (CDCl_3_): δ 134.2 (C-2), 138.1 (C-3), 131.3 (C-5), 113.6 (C-6), 116.2 (C-7), 131.8 (C-8), 129.2 (C-9), 107.5 (C-10), 163.8 (C-11), 109.6 (C-12), 145.1 (C-13), 16.1 (C-14), 207.4 (C-1′), 40.7 (C-2′), 18.8 (C-3′and C-4′), 56.8 (OCH_3_); HREIMS (*m/z*): 282.1362 [C_17_H_18_N_2_O_2_ calcd. 282.1368]; EIMS *m/z* (%): 282 [M^+^] (94), 239 (100), 224 (8), 211 (6), 196 (17). 

*12-Valeryl-11-methoxy-3-methyl-β-carboline* (**14**). 14.1 mg; mp: 289-290 ^o^C; IR (CHCl_3_) ν_max_ cm^-1^: 3385.1 (indole N-H), 2928.1, 2861.5 (C-H), 1648.8 (ketone C=O), 1612.5, 1543.9, 1416.2 (aromatic C=C), 1152.2 (C-O); UV (MeOH) λ_max_ nm: 361.2, 321.8, 269.4, 232.6; ^1^H-NMR (CDCl_3_): δ 8.34 (1H, d, *J =* 5.3 Hz, H-5), 7.84 (1H, d, *J =* 5.3 Hz, H-6), 8.25 (1H, d, *J =* 8.7 Hz, H-9), 7.03 (1H, d, *J =* 8.7 Hz, H-10), 3.02 (3H, s, H-14), 3.16 (2H, t, *J =* 7.2 Hz, H-2′), 1.73 (2H, quintet, *J =* 7.2 Hz, H-3′), 1.45 (2H, sextet, *J =* 7.2 Hz, H-4′), 0.97 (3H, t, *J =* 7.2 Hz, H-5′), 4.10 (3H, s, OCH_3_), 11.26 (1H, br.s, indole N-H); ^13^C-NMR (CDCl_3_): δ 134.2 (C-2), 138.4 (C-3), 131.4 (C-5), 113.8 (C-6), 116.0 (C-7), 132.1 (C-8), 129.4 (C-9), 107.6 (C-10), 163.9 (C-11), 110.1 (C-12), 144.9 (C-13), 16.2 (C-14), 203.2 (C-1′), 45.0 (C-2′), 26.5 (C-3′), 22.6 (C-4′), 14.0 (C-5′), 56.7 (OCH_3_); HREIMS (*m/z*): 296.1514 [C_18_H_20_N_2_O_2_ calcd. 296.1525]; EIMS *m/z* (%): 296 [M^+^] (86), 267 (39), 254 (52), 239 (100), 224 (19), 211 (2), 196 (10).

*12-Hexanoyl-11-methoxy-3-methyl-β-carboline* (**15**). 18.4 mg; mp: 292-293 ^o^C; IR (CHCl_3_) ν_max_ cm^-1^: 3385.0 (indole N-H), 2924.3, 2854.1 (C-H), 1648.8 (ketone C=O), 1614.5, 1546.4, 1460.0 (aromatic C=C), 1151.8 (C-O); UV (MeOH) λ_max_ nm: 361.2, 321.5, 269.3, 232.8; ^1^H-NMR (CDCl_3_):δ 8.35 (1H, d, *J =* 5.4 Hz, H-5), 7.86 (1H, d, *J =* 5.4 Hz, H-6), 8.20 (1H, d, *J =* 8.6 Hz, H-9), 6.95 (1H, d, *J =* 8.6 Hz, H-10), 2.97 (3H, s, H-14), 3.15 (2H, t, *J =* 7.3 Hz, H-2′), 1.75 (2H, quintet, *J =* 7.3 Hz, H-3′), 1.40 (4H, m, H-4′ and H-5′), 0.94 (3H, t, *J =* 7.3 Hz, H-6′), 4.07 (3H, s, OCH_3_), 11.25 (1H, br.s, indole N-H); ^13^C-NMR (CDCl_3_): δ 134.3 (C-2), 138.0 (C-3), 130.6 (C-5), 113.8 (C-6), 116.0 (C-7), 131.7 (C-8), 129.3 (C-9), 107.6 (C-10), 163.8 (C-11), 109.9 (C-12), 145.0 (C-13), 16.1 (C-14), 203.0 (C-1′), 44.2 (C-2′), 23.2 (C-3′), 31.1 (C-4′), 22.9 (C-5′), 14.2 (C-6′), 56.8 (OCH_3_); HREIMS (*m/z*): 310.1690 [C_19_H_22_N_2_O_2_ calcd. 310.1681]; EIMS *m/z* (%): 310 [M^+^] (26), 267 (6), 254 (11), 239 (100), 224 (12), 211 (6), 196 (8).

*12-Heptanoyl-11-methoxy-3-methyl-β-carboline* (**16**). 18.0 mg; mp: 296-297 ^o^C; IR (CHCl_3_) ν_max_ cm^-1^: 3384.5 (indole N-H), 2926.1, 2857.4 (C-H), 1648.2 (ketone C=O), 1608.2, 1542.9, 1455.2 (aromatic C=C), 1151.2 (C-O); UV (MeOH) λ_max_ nm: 361.8, 321.2, 269.3, 232.1; ^1^H-NMR (CDCl_3_): δ 8.35 (1H, d, *J =* 4.7 Hz, H-5), 7.85 (1H, d, *J =* 4.7 Hz, H-6), 8.27 (1H, d, *J =* 8.7 Hz, H-9), 7.02 (1H, d, *J =* 8.7 Hz, H-10), 3.05 (3H, s, H-14), 3.15 (2H, t, *J =* 7.3 Hz, H-2′), 1.74 (2H, quintet, *J =* 7.3 Hz, H-3′), 1.40 (2H, quintet, *J =* 7.3 Hz, H-4′), 1.34 (4H, m, H-5′ and H-6′), 0.89 (3H, t, *J =* 7.3 Hz, H-7′), 4.11 (3H, s, OCH_3_), 11.17 (1H, br.s, indole N-H); ^13^C-NMR (CDCl_3_): δ 134.2 (C-2), 137.9 (C-3), 131.0 (C-5), 114.0 (C-6), 115.7 (C-7), 131.8 (C-8), 129.4 (C-9), 107.5 (C-10), 163.8 (C-11), 110.0 (C-12), 145.1 (C-13), 16.0 (C-14), 203.4 (C-1′), 44.3 (C-2′), 24.1 (C-3′), 30.2 (C-4′), 32.2 (C-5′), 23.2 (C-6′), 14.4 (C-7′), 56.9 (OCH_3_); HREIMS (*m/z*): 324.1826 [C_20_H_24_N_2_O_2_ calcd. 324.1838]; EIMS *m/z* (%): 324 [M^+^] (84), 267 (53), 254 (66), 239 (100), 224 (12), 211 (7), 196 (8).

*12-Capryloyl-11-methoxy-3-methyl-β-carboline* (**17**). 18.3 mg; mp: 296-297 ^o^C; IR (CHCl_3_) ν_max_ cm^-1^: 3382.9 (indole N-H), 2923.9, 2854.5 (C-H), 1647.1 (ketone C=O), 1612.1, 1545.6, 1458.1 (aromatic C=C), 1153.2 (C-O); UV (MeOH) λ_max_ nm: 361.2, 321.5, 269.3, 232.4; ^1^H-NMR (CDCl_3_): δ 8.35 (1H, d, *J =* 5.2 Hz, H-5), 7.83 (1H, d, *J =* 5.2 Hz, H-6), 8.25 (1H, d, *J =* 8.7 Hz, H-9), 7.01 (1H, d, *J =* 8.7 Hz, H-10), 3.02 (3H, s, H-14), 3.16 (2H, t, *J =* 7.3 Hz, H-2′), 1.74 (2H, quintet, *J =* 7.3 Hz, H-3′), 1.37 (2H, quintet, *J =* 7.3 Hz, H-4′), 1.30 (6H, m, H-5′, H-6′ and H-7′), 0.86 (3H, t, *J =* 7.1 Hz, H-8′), 4.12 (3H, s, OCH_3_), 11.23 (1H, br.s, indole N-H); ^13^C-NMR (CDCl_3_): δ 134.2 (C-2), 137.7 (C-3), 130.4 (C-5), 114.0 (C-6), 115.7 (C-7), 132.3 (C-8), 129.5 (C-9), 107.8 (C-10), 163.9 (C-11), 110.2 (C-12), 145.1 (C-13), 15.7 (C-14), 203.1 (C-1′), 45.4 (C-2′), 24.3 (C-3′), 29.4 (C-4′)*, 29.3 (C-5′)*, 31.8 (C-6′), 22.6 (C-7′), 14.1 (C-8′), 56.9 (OCH_3_) (* values may be interchanged); HREIMS (*m/z*): 338.1988 [C_21_H_26_N_2_O_2_ calcd. 338.1994]; EIMS *m/z* (%): 338 [M^+^] (70), 267 (18), 254 (48), 239 (100), 224 (10), 211 (5), 196 (5). 

*10-Propionyl-11-hydroxy-3-methyl-β-carboline* (**18**). 28.5 mg; mp: 322-323 ^o^C; IR (CHCl_3_) ν_max_ cm^-1^: 3378.2 (OH and indole N-H), 2932.3, 2845.2 (C-H), 1646.8 (ketone C=O), 1612.1, 1535.3, 1443.1 (aromatic C=C), 1153.4 (C-O); UV (MeOH) λ_max_ nm: 347.2, 340.4, 275.2, 236.3; ^1^H-NMR (CD_3_OD): Table 2; ^13^C-NMR (CD_3_OD): Table 3; HREIMS (*m/z*): 254.1070 [C_15_H_14_N_2_O_2_ calcd. 254.1055]; EIMS *m/z* (%): 254 [M^+^] (79), 236 (9), 225 (100), 197 (6). 

*10-Butyryl-11-hydroxy-3-methyl-β-carboline* (**19**). 8.8 mg; mp: 323-324 ^o^C; IR (CHCl_3_) ν_max_ cm^-1^: 3379.5 (OH and indole N-H), 2954.3, 2863.1 (C-H), 1646.6 (ketone C=O), 1608.7, 1536.5, 1432.1 (aromatic C=C), 1158.3 (C-O); UV (MeOH) λ_max_ nm: 347.4, 340.2, 275.3, 236.5; ^1^H-NMR (CD_3_OD): δ 8.22 (1H, d, *J =* 5.6 Hz, H-5), 8.08 (1H, d, *J =* 5.6 Hz, H-6), 8.89 (1H, s, H-9), 6.94 (1H, s, H-12), 2.82 (3H, s, H-14), 3.20 (2H, t, *J =* 7.3 Hz, H-2′), 1.85 (2H, sextet, *J =* 7.4 Hz, H-3′), 1.08 (3H, t, *J =* 7.3 Hz, H-4′); ^13^C-NMR (CD_3_OD): δ 137.2 (C-2), 142.3 (C-3), 139.2 (C-5), 113.6 (C-6), 115.7 (C-7), 131.3 (C-8), 127.2 (C-9), 116.2 (C-10), 164.2 (C-11), 98.5 (C-12), 147.9 (C-13), 19.3 (C-14), 208.0 (C-1′), 38.2 (C-2′), 18.7 (C-3′), 14.3 (C-4′); HREIMS (*m/z*): 268.1220 [C_16_H_16_N_2_O_2_ calcd. 268.1212]; EIMS *m/z* (%): 268 [M^+^] (28), 253 (7), 240 (20), 225 (100), 197 (4). 

*10-Isobutyryl-11-hydroxy-3-methyl-β-carboline* (**20**). 20.2 mg; mp: 326-327 ^o^C; IR (CHCl_3_) ν_max_ cm^-1^: 3378.2 (OH and indole N-H), 2925.1, 2854.9 (C-H), 1647.2 (ketone C=O), 1608.1, 1537.1, 1458.3 (aromatic C=C), 1158.2 (C-O); UV (MeOH) λ_max_ nm: 347.5, 340.2, 275.1, 236.4; ^1^H-NMR (CD_3_OD): δ 8.18 (1H, d, *J =* 5.4 Hz, H-5), 7.97 (1H, d, *J =* 5.4 Hz, H-6), 8.83 (1H, s, H-9), 6.91 (1H, s, H-12), 2.77 (3H, s, H-14), 3.93 (1H, septet, *J =* 6.8 Hz, H-2′), 1.29 (6H, d, *J =* 6.8 Hz, H-3′ and H-4′); ^13^C-NMR (CD_3_OD): δ 137.4 (C-2), 141.8 (C-3), 138.7 (C-5), 114.2 (C-6), 115.0 (C-7), 131.6 (C-8), 127.4 (C-9), 116.2 (C-10), 165.4 (C-11), 98.9 (C-12), 148.3 (C-13), 18.8 (C-14), 212.0 (C-1′), 35.9 (C-2′), 20.1 (C-3′ and C-4′); HREIMS (*m/z*): 268.1218 [C_16_H_16_N_2_O_2_ calcd. 268.1212]; EIMS *m/z* (%): 268 [M^+^] (97), 253 (12), 225 (100), 197 (4).

*10-Hexanoyl-11-hydroxy-3-methyl-β-carboline* (**21**). 14.2 mg; mp: 327-328 ^o^C; IR (CHCl_3_) ν_max_ cm^-1^: 3373.2 (OH and indole N-H), 2923.9, 2854.5 (C-H), 1645.2 (ketone C=O), 1610.8, 1536.6, 1461.9 (aromatic C=C), 1199.6 (C-O); UV (MeOH) λ_max_ nm: 347.6, 340.0, 275.0, 236.2; ^1^H-NMR (CD_3_OD): δ 8.20 (1H, d, *J =* 5.2 Hz, H-5), 8.00 (1H, d, *J =* 5.2 Hz, H-6), 8.83 (1H, s, H-9), 6.91 (1H, s, H-12), 2.78 (3H, s, H-14), 3.20 (2H, t, *J =* 7.3 Hz, H-2′), 1.80 (2H, quintet, *J =* 7.3 Hz, H-3′), 1.43 (4H, m, H-4′ and H-5′), 0.95 (3H, t, *J =* 7.3 Hz, H-6′); ^13^C-NMR (CD_3_OD): δ 137.4 (C-2), 142.5 (C-3), 139.5 (C-5), 113.8 (C-6), 115.7 (C-7), 131.2 (C-8), 127.3 (C-9), 116.4 (C-10), 164.6 (C-11), 98.5 (C-12), 147.9 (C-13), 19.5 (C-14), 208.1 (C-1′), 38.9 (C-2′), 25.8 (C-3′), 32.6 (C-4′), 23.6 (C-5′), 14.3 (C-6′); HREIMS (*m/z*): 296.1518 [C_18_H_20_N_2_O_2_ calcd. 296.1525]; EIMS *m/z* (%): 296 [M^+^] (34), 278 (10), 253 (17), 240 (25), 225 (100), 197 (6).

*10-Heptanoyl-11-hydroxy-3-methyl-β-carboline* (**22**). 8.6 mg; mp: 329-330 ^o^C; IR (CHCl_3_) ν_max_ cm^-1^: 3375.4 (OH and indole N-H), 2924.2, 2856.1 (C-H), 1646.4 (ketone C=O), 1608.7, 1536.2, 1422.7 (aromatic C=C), 1182.1 (C-O); UV (MeOH) λ_max_ nm: 347.1, 339.9, 275.1, 236.3; ^1^H-NMR (CD_3_OD): δ 8.17 (1H, d, *J =* 5.7 Hz, H-5), 7.97 (1H, d, *J =* 5.7 Hz, H-6), 8.88 (1H, s, H-9), 6.92 (1H, s, H-12), 2.85 (3H, s, H-14), 3.07 (2H, t, *J =* 7.3 Hz, H-2′), 1.72 (2H, quintet, *J =* 7.3 Hz, H-3′), 1.37 (2H, quintet, *J =* 7.3 Hz, H-4′), 1.27 (4H, m, H-5′ and H-6′), 0.81 (3H, t, *J =* 7.3 Hz, H-7′); ^13^C-NMR (CD_3_OD): δ 137.5 (C-2), 142.3 (C-3), 139.5 (C-5), 113.7 (C-6), 115.8 (C-7), 131.1 (C-8), 127.2 (C-9), 116.3 (C-10), 164.3 (C-11), 98.4 (C-12), 147.6 (C-13), 19.4 (C-14), 207.9 (C-1′), 38.4 (C-2′), 24.4 (C-3′), 29.2 (C-4′), 31.9 (C-5′), 22.9 (C-6′), 14.7 (C-7′); HREIMS (*m/z*): 310.1673 [C_19_H_22_N_2_O_2_ calcd. 310.1681]; EIMS *m/z* (%): 310 [M^+^] (79), 292 (55), 253 (57), 240 (35), 225 (100), 197 (10). 

*10,12-Diacetyl-11-hydroxy-3-methyl-β-carboline* (**23**). 17.0 mg; mp: 328-329 ^o^C; IR (CHCl_3_) ν_max_ cm^‑1^: 3384.2 (OH and indole N-H), 2928.0, 2850.1 (C-H), 1644.2 (ketone C=O), 1582.4, 1494.6, 1426.5 (aromatic C=C), 1189.7 (C-O); UV (MeOH) λ_max_ nm: 364.5, 279.7, 230.2; ^1^H-NMR (CDCl_3_): δ 8.40 (1H, d, *J =* 5.3 Hz, H-5), 7.79 (1H, d, *J =* 5.3 Hz, H-6), 8.69 (1H, s, H-9), 2.88 (3H, s, H-14), 2.78 (3H, s, H-2′), 2.84 (3H, s, H-2′′), 14.67 (1H, s, OH), 11.33 (1H, br.s, indole N-H); ^13^C-NMR (CDCl_3_): δ 135.1 (C-2), 140.9 (C-3), 137.9 (C-5), 112.9 (C-6), 114.5 (C-7), 130.2 (C-8), 130.5 (C-9), 114.6 (C-10), 166.9 (C-11), 109.0 (C-12), 146.6 (C-13), 18.5 (C-14), 206.6 (C-1′), 32.2 (C-2′), 203.3 (C-1′′), 36.9 (C-2′′); HREIMS (*m/z*): 282.1012 [C_16_H_14_N_2_O_3_ calcd. 282.1004]; EIMS *m/z* (%): 282 [M^+^] (91), 267 (100), 251 (18), 249 (32), 239 (4), 237 (18).

*10,12-Dipropionyl-11-hydroxy-3-methyl-β-carboline* (**24**). 16.6 mg; mp: 330-331 ^o^C; IR (CHCl_3_) ν_max_ cm^-1^: 3383.2 (OH and indole N-H), 2927.2, 2851.0 (C-H), 1646.1 (ketone C=O), 1580.4, 1493.8, 1461.3 (aromatic C=C), 1189.2 (C-O); UV (MeOH) λ_max_ nm: 366.2, 279.8, 230.2; ^1^H-NMR (CD_3_OD): Table 2; ^13^C-NMR (CD_3_OD): Table 3; HREIMS (*m/z*): 310.1325 [C_18_H_18_N_2_O_3_ calcd. 310.1317]; EIMS *m/z* (%): 310 [M^+^] (100), 292 (12), 281 (98), 253 (5), 251 (19), 237 (24). 

*10,12-Dibutyryl-11-hydroxy-3-methyl-β-carboline* (**25**). 15.5 mg; mp: 331-332 ^o^C; IR (CHCl_3_) ν_max_ cm^-1^: 3383.5 (OH and indole N-H), 2926.5, 2856.5 (C-H), 1646.2 (ketone C=O), 1580.2, 1494.3, 1416.2 (aromatic C=C), 1188.5 (C-O); UV (MeOH) λ_max_ nm: 366.2, 279.7, 230.0; ^1^H-NMR (CDCl_3_): δ 8.41 (1H, d, *J =* 5.3 Hz, H-5), 7.82 (1H, d, *J =* 5.3 Hz, H-6), 8.69 (1H, s, H-9), 2.87 (3H, s, H-14), 3.14 (2H, t, *J =* 7.3 Hz, H-2′), 1.88 (2H, sextet, *J =* 7.3 Hz, H-3′), 1.08 (3H, t, H-4′), 3.24 (2H, t, *J =* 7.3 Hz, H-2′′), 1.79 (2H, sextet, *J =* 7.3 Hz, H-3′′), 1.04 (3H, t, *J =* 7.3 Hz, H-4′′), 14.74 (1H, s, OH), 11.28 (1H, br.s, indole N-H); ^13^C-NMR (CDCl_3_): δ 135.2 (C-2), 141.2 (C-3), 137.7 (C-5), 112.8 (C-6), 114.6 (C-7)*, 130.6 (C-8 and C-9), 114.8 (C-10)*, 167.0 (C-11), 108.9 (C-12), 146.7 (C-13), 18.5 (C-14), 206.7 (C-1′), 40.3 (C-2′), 18.3 (C-3′), 14.0 (C-4′)**, 203.1 (C-1′′), 47.2 (C-2′′), 17.6 (C-3′′), 13.9 (C-4′′)** (*,** values may be interchanged); HREIMS (*m/z*): 338.1625 [C_20_H_22_N_2_O_3_ calcd. 338.1630]; EIMS *m/z* (%): 338 [M^+^] (63), 323 (10), 295 (100), 282 (30), 277 (24), 267 (16), 251 (16), 237 (22). 

*10,12-Diisobutyryl-11-hydroxy-3-methyl-β-carboline* (**26**). 12.6 mg; mp: 333-334 ^o^C; IR (CHCl_3_) ν_max_ cm^-1^: 3385.1 (OH and indole N-H), 2926.0, 2854.0 (C-H), 1645.1 (ketone C=O), 1581.2, 1494.5, 1455.2 (aromatic C=C), 1187.2 (C-O); UV (MeOH) λ_max_ nm: 365.8, 279.8, 230.1; ^1^H-NMR (CDCl_3_): δ 8.41 (1H, d, *J =* 5.3 Hz, H-5), 7.82 (1H, d, *J =* 5.3 Hz, H-6), 8.75 (1H, s, H-9), 2.88 (3H, s, H-14), 3.89 (1H, septet, *J =* 7.3 Hz, H-2′), 1.33 (6H, d, *J =* 7.3 Hz, H-3′ and H-4′), 3.98 (1H, septet, *J =* 7.3 Hz, H-2′′), 1.26 (6H, d, *J =* 7.3 Hz, H-3′′′and H-4′′), 14.71 (1H, s, OH), 11.41 (1H, br.s, indole N-H); ^13^C-NMR (CDCl_3_): δ 135.1 (C-2), 141.1 (C-3), 137.8 (C-5), 112.9 (C-6), 114.4 (C-7), 130.2 (C-8), 130.7 (C-9), 114.5 (C-10), 166.9 (C-11), 108.8 (C-12), 146.5 (C-13), 18.6 (C-14), 211.8 (C-1′), 36.8 (C-2′), 19.3 (C-3′ and C-4′), 207.2 (C-1′′), 40.4 (C-2′′), 19.1 (C-3′′ and ′C-4′′); HREIMS (*m/z*): 338.1644 [C_20_H_22_N_2_O_3_ calcd. 338.1630]; EIMS *m/z* (%): 338 [M^+^] (36), 295 (100), 277 (45), 251 (22), 237 (26). 

*10,12-Divaleryl-11-hydroxy-3-methyl-β-carboline* (**27**). 25.5 mg; mp: 332-333 ^o^C; IR (CHCl_3_) ν_max_ cm^-1^: 3383.0 (OH and indole N-H), 2927.4, 2862.8 (C-H), 1644.2 (ketone C=O), 1582.4, 1495.3, 1418.7 (aromatic C=C), 1199.8 (C-O); UV (MeOH) λ_max_ nm: 364.8, 279.8, 230.0; ^1^H-NMR (CDCl_3_): δ 8.43 (1H, d, *J =* 5.3 Hz, H-5), 7.89 (1H, d, *J =* 5.3 Hz, H-6), 8.74 (1H, s, H-9), 2.93 (3H, s, H-14), 3.16 (2H, t, *J =* 7.3 Hz, H-2′), 1.80 (2H, quintet, *J =* 7.3 Hz, H-3′), 1.49 (2H, sextet, *J =* 7.3 Hz, H-4′), 0.99 (3H, t, *J =* 7.3 Hz, H-5′), 3.25 (2H, t, *J =* 7.3 Hz, H-2′′), 1.74 (2H, quintet, *J =* 7.3 Hz, H-3′′), 1.45 (2H, sextet, *J =* 7.3 Hz, H-4′′), 0.96 (3H, t, *J =* 7.3 Hz, H-5′′), 14.75 (1H, s, OH), 11.40 (1H, br.s, indole N-H); ^13^C-NMR (CDCl_3_): δ 135.2 (C-2), 141.0 (C-3), 137.7 (C-5), 112.8 (C-6), 114.6 (C-7), 130.0 (C-8), 130.6 (C-9), 114.6 (C-10), 166.9 (C-11), 109.0 (C-12), 146.6 (C-13), 18.5 (C-14), 206.8 (C-1′), 38.1 (C-2′), 26.3 (C-3′), 22.5 (C-4′)*, 14.0 (C-4′)**, 203.3 (C-1′′), 44.9 (C-2′′), 26.9 (C-3′′), 22.4 (C-4′′)*, 13.9 (C-5′′)** (*,** values may be inter-changed); HREIMS (*m/z*): 366.1935 [C_22_H_26_N_2_O_3_ calcd. 366.1943]; EIMS *m/z* (%): 366 [M^+^] (20), 348 (24), 337 (77), 324 (25), 309 (100), 295 (22), 282 (16), 267 (20), 251 (18), 237 (25).

*10,12-Dihexanoyl-11-hydroxy-3-methyl-β-carboline* (**28**). 25.1 mg; mp: 335-336 ^o^C; IR (CHCl_3_) ν_max_ cm^-1^: 3384.2 (OH and indole N-H), 2924.6, 2855.2 (C-H), 1645.2 (ketone C=O), 1581.2, 1494.8, 1456.3 (aromatic C=C), 1189.2 (C-O); UV (MeOH) λ_max_ nm: 364.9, 279.6, 230.2; ^1^H-NMR (CDCl_3_): δ 8.40 (1H, d, *J =* 5.2 Hz, H-5), 7.81 (1H, d, *J =* 5.2 Hz, H-6), 8.70 (1H, s, H-9), 2.89 (3H, s, H-14), 3.10 (2H, t, *J =* 7.3 Hz, H-2′), 1.81 (2H, quintet, *J =* 7.3 Hz, H-3′), 1.36 (8H, m, H-4′, H-5′, H-4′′′and H-5′′), 0.93 (3H, t, *J =* 6.8 Hz, H-6′), 3.21 (2H, t, *J =* 7.3 Hz, H-2′′), 1.74 (2H, quintet, *J =* 7.3 Hz, H-3′′), 0.91 (3H, t, *J =* 6.8 Hz, H-6′′), 14.69 (1H, s, OH), 11.31 (1H, br.s, indole N-H); ^13^C-NMR (CDCl_3_): δ 135.0 (C-2), 141.1 (C-3), 137.9 (C-5), 112.8 (C-6), 114.5 (C-7), 130.0 (C-8), 130.6 (C-9), 114.7 (C-10), 167.0 (C-11), 108.9 (C-12), 146.4 (C-13), 18.4 (C-14), 206.6 (C-1′), 38.3 (C-2′), 25.7 (C-3′), 32.9 (C-4′)*, 23.8 (C-5′)**, 14.2 (C-6′)^ψ^, 203.5 (C-1′′), 45.1 (C-2′′), 25.5 (C-3′′), 32.8 (C-4′′)*, 23.7 (C-5′′)**, 14.1 (C-6′′)^ψ^ (*,**,^Ψ^ values may be inter-changed); HREIMS (*m/z*): 394.2244 [C_24_H_30_N_2_O_3_ calcd. 394.2256]; EIMS *m/z* (%): 394 [M^+^] (100), 376 (32), 351 (87), 338 (6), 323 (98), 295 (30), 282 (19), 267 (13), 251 (21), 237 (18).

*10,12-Diheptanoyl-11-hydroxy-3-methyl-β-carboline* (**29**). 27.1 mg; mp: 336-337 ^o^C; IR (CHCl_3_) ν_max_ cm^-1^: 3383.8 (OH and indole N-H), 2925.3, 2855.4 (C-H), 1644.2 (ketone C=O), 1580.0, 1495.5, 1450.1 (aromatic C=C), 1201.1 (C-O); UV (MeOH) λ_max_ nm: 366.8, 284.6, 231.8; ^1^H-NMR (CDCl_3_): δ 8.43 (1H, d, *J =* 5.3 Hz, H-5), 7.85 (1H, d, *J =* 5.3 Hz, H-6), 8.72 (1H, s, H-9), 2.92 (3H, s, H-14), 3.15 (2H, t, *J =* 7.3 Hz, H-2′), 1.82 (2H, quintet, *J =* 7.3 Hz, H-3′), 1.45 (2H, m, H-4′), 1.34 (8H, m, H-5′, H-6′, H-5′′ and ′H-6′′), 0.90 (3H, t, *J =* 6.8 Hz, H-7′), 3.25 (2H, t, *J =* 7.3 Hz, H-2′′), 1.75 (2H, quintet, *J =* 7.3 Hz, H-3′′), 1.42 (2H, m, H-4′′), 0.89 (3H, t, *J =* 6.8 Hz, H-7′′), 14.75 (1H, s, OH), 11.38 (1H, br.s, indole N-H); ^13^C-NMR (CDCl_3_): δ 135.1 (C-2), 140.9 (C-3), 137.9 (C-5), 112.7 (C-6), 114.5 (C-7)*, 129.9 (C-8), 130.5 (C-9), 114.6 (C-10)*, 166.9 (C-11), 108.7 (C-12), 146.5 (C-13), 18.4 (C-14), 206.8 (C-1′), 38.4 (C-2′), 24.1 (C-3′), 29.1 (C-4′)**, 31.8 (C-5′)^ψ^, 22.6 (C-6′)^ψψ^, 14.1 (C-7′)^†^, 203.3 (C-1′′), 45.3 (C-2′′), 24.7 (C-3′′), 28.9 (C-4′′)**, 31.6 (C-5′′)^ψ^, 22.5 (C-6′′)^ψψ^, 14.0 (C-7′′)^† ^(*,**,^Ψ^,^ΨΨ^,^†^ values may be interchanged); HREIMS (*m/z*): 422.2581 [C_26_H_34_N_2_O_3_ calcd. 422.2569]; EIMS *m/z* (%): 422 [M^+^] (100), 404 (25), 365 (57), 352 (24), 337 (94), 309 (2), 295 (25), 282 (27), 267 (37), 251 (24), 237 (28).

*10,12-Dicapryloyl-11-hydroxy-3-methyl-β-carboline* (**30**). 27.1 mg; mp: 339-340 ^o^C; IR (CHCl_3_) ν_max_ cm^-1^: 3381.0 (OH and indole N-H), 2925.8, 2858.3 (C-H), 1643.2 (ketone C=O), 1579.6, 1496.8, 1377.2 (aromatic C=C), 1202.8 (C-O); UV (MeOH) λ_max_ nm: 365.9, 283.8, 231.6; ^1^H-NMR (CDCl_3_): δ 8.41 (1H, d, *J =* 5.4 Hz, H-5), 7.82 (1H, d, *J =* 5.4 Hz, H-6), 8.71 (1H, s, H-9), 2.91 (3H, s, H-14), 3.15 (2H, t, *J =* 7.3 Hz, H-2′), 1.80 (2H, quintet, *J =* 7.3 Hz, H-3′), 1.43 (2H, m, H-4′), 1.30 (12H, m, H-5′, H-6′, H-7′, H-5′′′′H-6′′ and H-7′′), 0.88 (3H, t, *J =* 6.8 Hz, H-8′), 3.25 (2H, t, *J =* 7.3 Hz, H-2′′), 1.76 (2H, quintet, *J =* 7.3 Hz, H-3′′), 1.41 (2H, m, H-4′′), 0.87 (3H, t, *J =* 6.8 Hz, H-8′′), 14.75 (1H, s, OH), 11.36 (1H, br.s, indole N-H); ^13^C-NMR (CDCl_3_): δ 135.0 (C-2), 141.0 (C-3), 137.8 (C-5), 112.9 (C-6), 114.2 (C-7), 131.0 (C-8), 131.1 (C-9), 115.0 (C-10), 167.3 (C-11), 108.9 (C-12), 146.5 (C-13), 18.3 (C-14), 206.9 (C-1′), 38.5 (C-2′), 24.8 (C-3′), 29.3 (C-4′)*, 29.2 (C-5′)*, 31.8 (C-6′)**, 22.6 (C-7′ and C-7′′), 14.1 (C-8′), 203.2 (C-1′′), 45.3 (C-2′′), 24.1 (C-3′′), 29.2 (C-4′′)*, 29.1 (C-5′′)*, 31.7 (C-6′′)**, 14.1 (C-8′′) (*,** values may be interchanged); HREIMS (*m/z*): 450.2898 [C_28_H_38_N_2_O_3_ calcd. 450.2882]; EIMS *m/z* (%): 450 [M^+^] (98), 432 (43), 403 (6), 379 (83), 366 (51), 351 (100), 323 (2), 295 (32), 282 (26), 267 (32), 251 (14), 237 (21).

**Table 2:** ^1^H-NMR data of 10-propionyl (**3**), 12-propionyl (**11**), 10-propionyl-11-hydroxy (**18**) and 10,12-dipropionyl-11-hydroxy (**24**) analogues of harmine (**1**).

H	3	11	18	24
5	8.21 (d, 5.7)	8.35 (d, 5.5)	8.15 (d, 5.3)	8.42 (d, 5.3)
6	8.06 (d, 5.7)	7.82 (d, 5.5)	7.94 (d, 5.3)	7.87 (d, 5.3)
9	8.50 (s)	8.23 (d, 8.7)	8.81 (s)	8.72 (s)
10	-	6.99 (d, 8.7)	-	-
12	7.16 (s)	-	6.91 (s)	-
14	2.87 (s)	2.97 (s)	2.77 (s)	2.94 (s)
2′	3.06 (q, 7.3)	3.19 (q, 7.1)	3.20 (q,7.2)	3.23 (q, 7.3)
3′	1.18 (t, 7.3)	1.24 (t, 7.1)	1.25 (t, 7.2)	1.34 (t, 7.3)
OCH_3_	4.04 (s)	4.08 (s)	-	-
2′′	-	-	-	3.31 (q, 7.3)
3′′	-	-	-	1.25 (t, 7.3)
OH	-	-	Not observed	14.69 (s)
Indole N-H	Not observed	11.20 (br.s)	Not observed	11.40 (br.s)

*Note*: NMRs of **3** and **18** were recorded in CD_3_OD, whereas those of **11** and **24** were recorded in CDCl_3_. Assignments were established by interpretation of the ^1^H-NMR, HMQC, HMBC, ^1^H, ^1^H COSY, ^1^H,^1^H TOCSY, and *J*-resolved spectra. Values are in δ (ppm). Multiplicities and *J* values (in Hz) are in parentheses.

**Table 3:** ^13^C-NMR data of 10-propionyl (**3**), 12-propionyl (**11**), 10-propionyl-11-hydroxy (**18**) and 10,12-dipropionyl-11-hydroxy (**24**) analogues of harmine (**1**).

C	3	11	18	24
2	137.0	134.3	137.4	135.0
3	142.5	138.0	142.4	141.0
5	139.5	131.5	139.3	137.7
6	113.6	113.7	113.8	112.8
7	116.2	116.1	115.6	114.6
8	130.6	131.9	131.2	129.9
9	126.0	129.4	127.1	130.6
10	124.1	107.6	116.3	114.5
11	161.9	163.5	164.4	167.0
12	94.6	109.9	98.5	108.9
13	146.8	144.9	147.8	146.7
14	19.3	15.8	19.4	18.3
1′	204.2	203.4	208.2	206.7
2′	37.5	37.0	32.2	32.4
3′	9.4	9.2	8.9	9.2
OCH_3_	56.4	56.7	-	-
1′′	-	-	-	203.5
2′′	-	-	-	44.9
3′′	-	-	-	9.5

*Note*: NMRs of **3** and **18** were recorded in CD_3_OD, whereas those of **11** and **24** were recorded in CDCl_3_. Assignments were established by interpretation of the ^13^C-NMR (broad band decoupled and DEPT), HMQC, and HMBC spectra.
